# Guillain-Barré Syndrome Presenting With Unilateral Facial Nerve Palsy: A Rare Presentation

**DOI:** 10.7759/cureus.104776

**Published:** 2026-03-06

**Authors:** Fatima Alam, Suham Amin, Nazil Anwar

**Affiliations:** 1 Acute Medicine, Stockport NHS Foundation Trust, Stockport, GBR; 2 Acute Medicine, Stepping Hill Hospital, Stockport, GBR; 3 Acute Internal Medicine, Stockport NHS Foundation Trust, Stockport, GBR

**Keywords:** acute lower back pain, guillain-barré syndrome (gbs), intravenous immunoglobulin (ivig), rare clinical presentation, unilateral facial nerve palsy

## Abstract

Guillain-Barré syndrome (GBS) is an acute immune-mediated polyradiculoneuropathy typically presenting with symmetrical ascending limb weakness and areflexia. Cranial nerve involvement is recognized, most commonly affecting the facial nerves bilaterally. Unilateral facial nerve palsy, particularly in association with severe radicular lower back pain, is an atypical presentation and may lead to diagnostic uncertainty. We report the case of a 40-year-old woman with a background of papillary thyroid cancer who presented with severe lower back pain, progressive bilateral lower limb weakness, and acute left-sided lower motor neuron facial nerve palsy following a flu-like illness with diarrheal symptoms. Initial assessment focused on stroke and malignancy recurrence. Rapid neurological deterioration with bulbar symptoms and respiratory compromise prompted further investigation. Cerebrospinal fluid analysis demonstrated albuminocytological dissociation, and nerve conduction studies confirmed GBS. The patient required intensive care admission and was treated with intravenous immunoglobulin, resulting in significant clinical improvement. This case highlights a rare and atypical presentation of GBS and emphasizes the importance of recognizing asymmetrical cranial nerve involvement to facilitate early diagnosis and timely treatment.

## Introduction

Guillain-Barré syndrome (GBS) is an acute immune-mediated disorder of the peripheral nervous system and a leading cause of acute flaccid paralysis worldwide. It is most characterized by rapidly progressive, symmetrical ascending limb weakness, areflexia, and variable sensory and autonomic dysfunction [1,2]. Several subtypes have been identified based on electrophysiological and pathological features, including acute inflammatory demyelinating polyradiculoneuropathy (AIDP), acute motor axonal neuropathy (AMAN), acute motor-sensory axonal neuropathy (AMSAN), and Miller Fisher syndrome (MFS) [3].

Cranial nerves are affected in a significant proportion of patients with GBS. Bulbar palsy is the most common, followed by facial nerve involvement. Facial nerve palsy, when present, is typically bilateral and symmetrical [4,5]. Unilateral facial nerve palsy is rare (3-5%), with limited literature on the subject. Such atypical presentations can mimic more common neurological conditions such as acute stroke and idiopathic Bell’s palsy, resulting in diagnostic delay.

We report a case of GBS presenting with unilateral lower motor neuron facial nerve palsy, severe back pain, and rapidly progressive limb weakness, highlighting the diagnostic challenges and importance of early recognition.

## Case presentation

A 40-year-old woman presented to the emergency department with severe lower back pain, progressive bilateral lower limb weakness, and acute left-sided facial asymmetry. She was previously fully independent and normally mobile. Her past medical history included anxiety, depression, and papillary thyroid carcinoma treated with total thyroidectomy and radioiodine therapy 10 years earlier, resulting in acquired hypothyroidism. Her current and past medications included levothyroxine, vitamin D, collagen, biotin, and vitamin B12.

Two weeks before presentation, she discontinued her supplements following oncology advice due to abnormal thyroid function tests, with concerns regarding impaired levothyroxine absorption and possible cancer reactivation. Following cessation of supplements, she developed flu-like symptoms, including fever, myalgia, lethargy, and several episodes of diarrhoea. A few days later, she experienced sharp lower back pain and paraesthesia in her hands and feet, which gradually worsened. This was followed by gait instability, inability to stand without support, severe leg pain, urinary incontinence, and constipation. Twenty-four hours before presentation, her family noticed a left-sided facial droop and dysarthria. She was advised by her cancer specialist center to attend the emergency department.

On arrival, she was assessed via the hyper-acute stroke pathway. Initial neurological assessment showed normal sensations, left-sided lower motor neuron facial palsy, power of 4/5 in all limbs with normal tone, diminished bilateral ankle and knee reflexes with normal bilateral upper limb reflexes, and reduced anal tone on per-rectal examination.

Biochemical assessment revealed elevated thyroid-stimulating hormone at 37 mIU/L with low-normal free thyroxine, consistent with poorly controlled hypothyroidism. Other routine blood parameters were unremarkable (Table 1).

**Table 1:** Basic blood panel showing poorly controlled hypothyroidism. WCC = white cell count; MCV = mean corpuscular volume; INR = international normalized ratio; eGFR = estimated glomerular filtration rate; ALT = alanine aminotransferase; AST = alkaline phosphatase; GGT = gamma-glutamyl transferase; TSH = thyroid-stimulating hormone

Test	Result	Normal range
WCC (10^9^/L)	9.6	3.7–11.0
Hemoglobin (g/L)	115	115–165
MCV (fL)	75.2	76–104
Platelet (10^9^/L)	372	150–450
Neutrophil (10^9^/L)	7.0	1.7–7.5
Lymphocyte (10^9^/L)	1.9	1.0–4.5
INR	1.0	-
Sodium (mmol/L)	139	132–146
Potassium (mmol/L)	4.3	3.5–5.0
Urea (mmol/L)	3.7	2.5–8.5
Creatine (µmol/L)	78	45–100
eGFR (mL/minute)	75	>60
Bilirubin (µmol/L)	7	3–19
ALT (U/L)	51	0–31
ALP (U/L)	74	35–125
GGT (U/L)	35	2–35
Calcium (mmol/L)	0.47	2.15–2.60
T4 (pmol/L)	10.8	11.0–22.0
TSH (mU/L)	37	0.10–4.0

**Figure 1:**
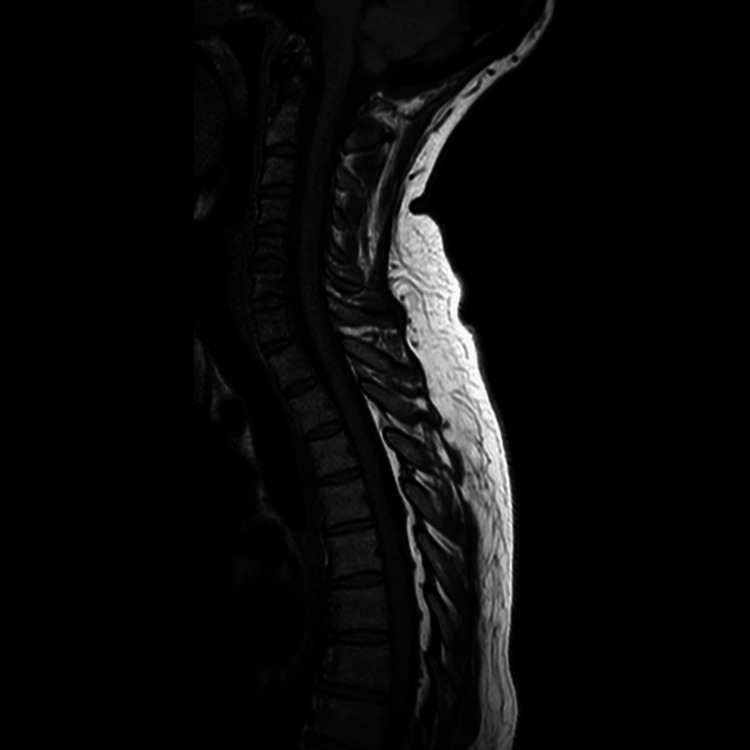
MRI of the cervical spine: normal with no signs of metastasis.

**Figure 2:**
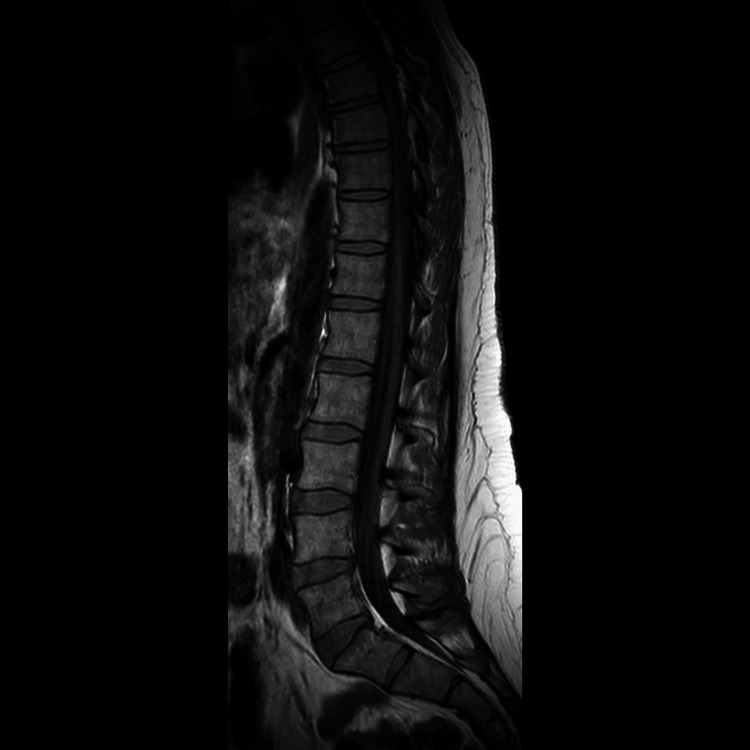
MRI of the thoraco-lumbar spine: normal with no signs of metastasis..

**Figure 3:**
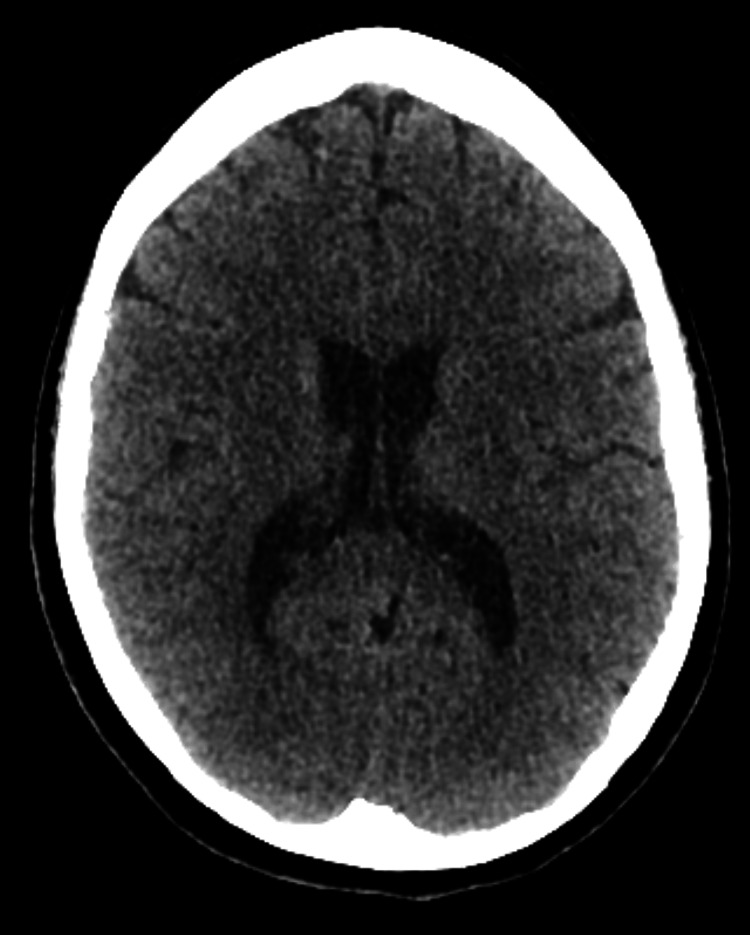
CT of the head: normal with no signs of stroke or intra-cerebral lesion.

Radiological assessment was unremarkable. MRI of the whole spine was done to rule out the possibility of metastasis (Figures 1, 2). CT of the thorax, abdomen, and pelvis was also unremarkable (Figure 3).

Within 24 hours of admission, the patient developed worsening lower limb weakness, severe limb pain, dysphagia, and worsening speech. Repeat neurological examination demonstrated a power of 4/5 with hypotonia and diminished reflexes in upper limbs, a power of 3/5 with marked hypotonia and absent reflexes in lower limbs, persistent unilateral facial nerve palsy with external ophthalmoplegia, and declining single-breath count.

Given the rapid progression, GBS was suspected. Cerebrospinal fluid analysis demonstrated albuminocytological dissociation, with elevated protein and normal white cell count (Table 2). Cultures were negative.

**Table 2:** Lumbar puncture analysis showing high protein. High protein in the CSF sample guided us in the diagnosis of GBS. WCC = white cell count; RBC = red blood cell count; CSF = cerebrospinal fluid; GBS = Guillain-Barré syndrome

Test	Result	Normal range
Protein (g/L)	1.52	0.18–0.45
Glucose (mmol/L)	4	2.5–3.5
Lactate	2	-
Gram stain	Negative	-
WCC (10^6^/L)	4	<3
RBC (10^6^/L)	513 (traumatic sample)	-
Culture	Negative	-

The patient deteriorated further with respiratory compromise, necessitating transfer to the intensive care unit. She was commenced on a five-day course of intravenous immunoglobulin (IVIG). Her neurological status improved significantly following treatment. Nerve conduction studies confirmed the diagnosis of GBS, demonstrating abnormalities in both peripheral nerves and the left facial nerve.

## Discussion

GBS commonly presents with symmetrical bilateral lower limb paralysis, dysautonomia, and areflexia [1]. There are four subtypes based on pathophysiology and histology. AIDP is mediated by T cells directed against myelin proteins. AMAN and AMSAN are caused by antibodies directed against the axonal sheath. MFS is also antibody-mediated and has predominant facial nerve involvement. Sensory involvement, as mentioned above in the AMSAN subtype, is known, but patients mostly present with peripheral paraesthesia [3]. Severe back pains along with urinary incontinence and constipation, as mentioned in this case, can mimic a cauda equina-like presentation, making it very challenging to reach the correct diagnosis and potentially delaying the treatment, leading to worse outcomes.

Studies have shown that cranial nerve involvement is not unknown. Limited literature on cranial nerve palsies and their association with GBS shows that bulbar palsy is the most common, followed by bilateral symmetrical facial nerve involvement (MFS) [4]. Unilateral facial nerve palsy is an uncommon manifestation. One study showed that only 3% of GBS cases with cranial nerve involvement had unilateral facial nerve palsy [4,5].

The pathophysiology underlying unilateral cranial nerve involvement and back pain with spinal symptoms remains unclear but may relate to focal immune-mediated nerve injury or asymmetric antiganglioside antibody distribution [3]. In this case, the unilateral facial palsy, severe back pain, and rapid progression initially mimicked stroke, malignancy recurrence, and spinal pathology, delaying diagnosis and leading to intensive care unit admission. Along with this diagnostic uncertainty, this case also highlights the difference between subtypes of the disease, i.e., AIDP versus MFS. Involvement of the facial nerve and ophthalmoplegia mimicked MFS, but lower limb weakness with unilateral facial nerve palsy favored the diagnosis of AIDP.

Early recognition and prompt administration of immunoglobulins are essential in reducing morbidity and preventing life-threatening complications such as respiratory failure and long-term paralysis [1].

## Conclusions

GBS typically presents with symmetrical limb weakness that may or may not be associated with symmetrical cranial nerve involvement. This case highlights the importance of considering the possibility of GBS in patients who present with atypical features such as unilateral lower motor neuron facial nerve palsy and severe back pain, along with typical flaccid paralysis. Awareness of such atypical and rare presentations is crucial to ensure early diagnosis and timely administration of immunoglobulins to prevent respiratory failure and long-term neurological disability.
